# Chinese Herbal Medicine for Irritable Bowel Syndrome: A Meta-Analysis and Trial Sequential Analysis of Randomized Controlled Trials

**DOI:** 10.3389/fphar.2021.694741

**Published:** 2021-07-27

**Authors:** Hui Zheng, Song Jin, Yin-Li Shen, Wen-Yan Peng, Kun Ye, Tai-Chun Tang, Jun Zhao, Min Chen, Zhi-Gang Li

**Affiliations:** ^1^ The Third Hospital, Acupuncture and Tuina School, Chengdu University of Traditional Chinese Medicine, Chengdu, China; ^2^ Department of Colorectal Diseases, Hospital of Chengdu University of Traditional Chinese Medicine, Chengdu, China; ^3^ Department of Rehabilitation, Hospital of Chengdu University of Traditional Chinese Medicine, Chengdu, China; ^4^ School of Acupuncture-Moxibustion and Tuina, Beijing University of Chinese Medicine, Beijing, China

**Keywords:** Chinese herbal medicine, irritable bowel syndrome, meta-analysis, trial sequential analysis, systematic review

## Abstract

**Purpose:** Chinese herbal medicine (CHM) is an important complementary and alternative therapy for the management of irritable bowel syndrome (IBS). Previous meta-analyses suggested that CHM is effective for IBS; nonetheless, its effectiveness is inconclusive owing to repeated significance testing. We aimed to examine the efficacy and safety of CHM for IBS through a meta-analysis and trial sequential analysis (TSA).

**Methods:** We searched OVID Medline, Embase, Cochrane Central Register of Controlled Trials, and Web of Science from January 1, 1980, to September 20, 2020. The primary outcome was adequate relief of global IBS symptoms. The secondary outcomes included relief of abdominal pain and treatment-related adverse events. The relative ratio (RR) and required information size (RIS) were calculated for each outcome.

**Results:** Ten trials recruiting 2,501 participants were included. Seven (70%) trials were at low risk of bias (RoB). Compared with placebo, CHM was associated with a significantly higher proportion of adequate relief of global IBS symptoms [RR 1.76 (95% confidence interval (95%CI), 1.33–2.33); *I* ^*2*^ = 81.1%; *p* < 0.001]. The RIS was 1,083 for the primary outcome, and the accrued information size was 1,716. The analysis of the relief of abdominal pain (three trials with 916 participants) showed similar results compared with placebo [RR 1.85 (95%CI, 1.59–2.14); *I* ^*2*^ = 0%; *p* < 0.001; RIS = 197 participants]. CHM was associated with a higher proportion of adverse events compared with placebo [RR 1.51 (95%CI, 1.14–2); *I* ^*2*^ = 0%; *p* = 0.004].

**Conclusion:** CHM was effective in relieving IBS symptoms but caused a higher adverse event rate than placebo. TSA analysis confirmed the findings with sufficient information size.

## Highlights

• Chinese herbal medicine (CHM) is widely used for patients with irritable bowel syndrome (IBS); however, its effectiveness is unconfirmed owing to repeated significance testing in previous meta-analyses.
• CHM was associated with a higher proportion of adequate relief of global IBS symptoms than placebo, and trial sequential analysis (TSA) showed that the results were with sufficient information size.
• CHM was associated with higher rates of adverse events compared with placebo, although most of the adverse events were mild without the need for additional care.
• Our meta-analysis and TSA analysis indicated that CHM might be a potential candidate for the treatment of IBS.

## Introduction

Irritable bowel syndrome (IBS) is a common clinical condition in gastroenterological clinics (Ford et al., 2017; Simrén et al., 2017; Sultan and Malhotra, 2017). Patients with IBS usually complained about changed bowel habits (constipation, diarrhea, or the mix of them), abdominal pain, and psychological disorders. IBS causes loss of working days, frequent visits to clinics, psychological disturbance, and low quality of life (Doshi et al., 2014; Tack et al., 2019; Black and Ford, 2020).

Many treatments are proposed for IBS (Mearin et al., 2016; Moayyedi et al., 2019; Lacy et al., 2021), and dietary interventions and pharmacological treatments (Chen et al., 2020; Lacy et al., 2021) are usually the primary choice. Owing to the complexity of the pathological mechanism of IBS, the dietary interventions and pharmacological treatments only worked for part of the IBS symptoms and achieved a small size of effect when compared with placebo (Carrasco-Labra et al., 2019; Chen et al., 2020).

When conventional pharmacological treatments failed to improve IBS symptoms, patients tend to pursue complementary and alternative medicines (CAMs) like herbal medicine and acupuncture (Jin et al., 2019). Chinese herbal medicine (CHM) is routinely practiced for functional gastrointestinal disorders in China, although the evidence for its effectiveness was still under investigation (Moayyedi et al., 2019). Several systematic reviews suggest that CHM is effective for the management of IBS, but the sample size of the included trials is commonly small, meaning that the conclusion might be based on underpowered trials (Bian et al., 2006; Shi et al., 2008; Bahrami et al., 2016; Dai et al., 2018; Tan et al., 2019; Yan et al., 2019; Zhou et al., 2019). Therefore, although the systematic reviews found that the design of currently published trials (from the year 2015–2020) had sufficient quality, the study power of current trials or meta-analyses was still questioned.

Meta-analyses will increase the likelihood of type I error, a phenomenon of multiplicity owing to repeated significance testing (Borm and Donders, 2009), when the meta-analyses are regularly updated with data from studies with small sample sizes (sparse data). Previous studies reported that one to three out of ten meta-analyses might be falsely reported as beneficial or harmful owing to the type I error (Borm and Donders, 2009; Imberger et al., 2016; Shah and Smith, 2020).

Based on the aforementioned facts, we conducted a systematic review with meta-analysis and a trial sequential analysis (TSA) of CHM for IBS, aiming to 1) examine whether CHM is effective for IBS after adjusting for the significance level and 2) explore whether new trials are warranted for specific outcomes (i.e., adequate relief of IBS symptoms and abdominal pain).

## Methods

### Study Source

OVID Medline, Embase, Cochrane Central Register of Controlled Trials, and Web of Science were searched from January 1, 1980, to September 20, 2020, without any language restrictions for randomized controlled trials (RCTs) that examined the efficacy of CHM in the management of IBS. Comprehensive search strategies for the databases are shown in the supplementary files (Supplementary Tables 1–4). The clinical registries (clinicaltrials.gov and www.chictr.org.cn) were also searched for RCTs that were completed but unpublished. We also searched previously published systematic reviews and read their reference lists to search for any missing RCTs.

### Study Selection

Two reviewers independently screened the retrieved articles, first at the abstract and title level and second at the full-text level.

#### Types of Studies

RCTs with the parallel design were included; those with crossover design were included if the results of the first phase (before the crossover of treatment arms) were separately reported.

#### Types of Participants

Adult participants with IBS were included. The diagnostic criteria should be developed based on the Rome criteria (Rome I, II, III, or IV) or criteria suggested by guidelines. RCTs were excluded for the simultaneous inclusion of both IBS and inflammatory bowel diseases unless the results of IBS were separately reported. We put no restrictions on underlying diseases (i.e., anxiety, depression, and other functional gastrointestinal disorders) and sex since the comorbidity of IBS with other disorders is common (Whitehead et al., 2002) and there is no evidence suggesting a difference in the effect of CHM caused by sex.

#### Types of Interventions and Controls

The experimental arm was CHM used as monotherapy or as adjunctive therapy; the control arm was placebo, active control (interventions recommended by guidelines (Mearin et al., 2016; Moayyedi et al., 2019; Lacy et al., 2021), or usual care. The active controls referred to antispasmodics (e.g., dicyclomine, hyoscine, and pinaverium), tricyclic antidepressants (e.g., amitriptyline), selective serotonin reuptake inhibitors (e.g., citalopram, fluoxetine, and paroxetine), 5-HT_3_ antagonists (e.g., alosetron, ramosetron, and cilansetron), 5-HT_4_ agonists (e.g., prucalopride), and guanylate cyclase-C agonists (e.g., linaclotide). RCTs with oral administration of CHM were included, and a minimum of 4 weeks in the treatment duration was required. Previous systematic reviews and our pilot search showed that most of the studies adopted a treatment duration of 4–8 weeks. To reduce heterogeneity caused by treatment duration and to exclude trials aiming to test an immediate effect of CHM, we required a minimum of 4 weeks in the treatment duration; considering that IBS is a chronic and refractory disease, we are not interested in the immediate effect of CHM on IBS. The preparation type of CHM was not restricted, and granules and decoctions were mostly used in the included RCTs.

#### Types of Outcomes

We included RCTs assessing any of the following outcomes: adequate relief of global IBS symptoms, relief of abdominal pain, or adverse events. The length of the follow-up period was unrestricted for this study.

Disagreement in the eligibility of RCTs was solved by group discussion and arbitrated by a third reviewer (HZ).

### Outcome Assessments

The primary outcome was adequate relief of global IBS symptoms, which was defined by asking participants directly whether they had achieved adequate relief of IBS related symptoms (abdominal pain, bowel movement disorders, and abdominal discomfort), by assessing symptoms through the Irritable Bowel Syndrome Severity Scoring System (IBS-SSS), having a change in IBS-SSS score for at least 75 points, or by adopting the Food and Drug Administration (FDA) criteria for IBS remission, a composite outcome including response to both abdominal pain and stool consistency. The developers of IBS-SSS stated that a change in the IBS-SSS score larger than 50 points indicates clinical improvement, but they did not report the cut-off point of the change in a score that indicates adequate relief of IBS symptoms. The patients with adequate relief of IBS symptoms reported the changes in the IBS-SSS score between 63.3 and 94.2 points (Passos et al., 2009; Dale et al., 2019), so we adopted a change for at least 75 points as indication of adequate relief of IBS symptoms based on the pooled results of the published studies. When the primary outcome was measured at several follow-up time points, we selected the time point that was assessed at the end of treatment.

The secondary outcomes included relief of abdominal pain and treatment-related adverse events. The relief of abdominal pain could be measured by visual analog scale (VAS) or other pain intensity rating scales like numeric rating scale, and a mean reduction for at least 30% compared with baseline was considered as relief of abdominal pain (Chang et al., 2016; Kerckhove et al., 2017; Lembo et al., 2020). When the relief of abdominal pain was not reported, we contacted the authors to ask for relevant data to make the judgment.

### Data Abstraction

Two reviewers extracted data from the included RCTs using standardized extraction forms. They firstly recorded trial characteristics, names of the first author, year of publication, study designs, sample sizes, mean ages of the included participants, proportions of females, and the subtype of IBS. Details of CHM intervention and controls were secondly extracted: the types of CHM (standardized or individualized formulation), dosage and administration frequency of CHM, types of controls, and treatment durations. The standardized CHM referred to an herbal formula with fixed composition and dosage, while the composition of the individualized CHM would be changed according to a participant’s syndrome differentiation. Outcome parameters were thirdly extracted: the name of the allocated arm, the number of participants achieving the predefined outcomes, and the number of participants in each arm. Disagreement in data abstraction was also solved by group discussion and arbitrated by a third reviewer. When data were unavailable from the retrieved articles, we tried to contact the authors to acquire the study data.

### Risk of Bias Assessment

The risk of bias (RoB) of the included trials was assessed by using the second version of the Cochrane RoB tool (RoB 2.0) (Sterne et al., 2019). The RoB 2.0 was updated based on the previous version of the Cochrane RoB tool released in 2008, in which five domains focusing on different aspects of trial design, conduct, and reporting were assessed. Within each domain, several signaling questions would be answered until the RoB level of a trial was determined. Each domain of a trial was rated with low RoB, high RoB, or some concerns; and an overall RoB was lastly rated for the trial. The RoB assessment was completed through an Excel template—which could be acquired at https://www.riskofbias.info—by two independent reviewers; discrepancy in the RoB assessment was solved by discussion.

### Data Synthesis

Conventional meta-analysis was performed by using the meta-package (R 4.0.2, www.r-project.org) to compare CHM with placebo or active controls. The results were firstly pooled through a random-effect model by using the DerSimonian-Laird method. Relative ratios (RRs) and their corresponding 95% confidence intervals (95%CIs) were calculated by the model; when the 95%CI of a pooled result excluded the null value, we considered a statistically significant difference between CHM and a control. Heterogeneity was secondly assessed according to the Cochrane handbook (version 5.1): 0–40%, might not be important; 30–60%, may represent moderate heterogeneity; 50–90%, may represent substantial heterogeneity; 75–100%: considerable heterogeneity.

Publication bias was assessed using contour-enhanced forest plots. We also performed trim-and-fill analysis to recalculate the pooled effect size of CHM with adjustment for publication bias.

Leave-one-out analysis was performed as sensitivity analysis, which assesses the impact of a specific study on the meta-analysis through removing one study at a time from the meta-analysis. Subgroup analysis was performed by including only participants with diarrhea-predominant IBS, and it was performed on adequate relief of global IBS symptoms and adverse event rate since the number of studies was small in assessing relief of abdominal pain.

TSA was performed to estimate the required information size (RIS) needed for each comparison for a specific outcome. The TSA analysis is analogous to sample size calculation in RCTs with several interim analyses. We assumed that a 20% difference in the rates of the outcomes between CHM and placebo was clinically meaningful in estimating RISs, as previously published systematic reviews all showed a difference ≥20% in the responder rates between CHM interventions and controls (Li et al., 2015; Dai et al., 2018; Tan et al., 2019; Zhou et al., 2019). To ensure the robustness of the TSA analysis, we additionally estimated the RISs by setting the difference at 15 and 10% for the primary outcome, respectively. For the safety outcome, the adverse event rate, we considered a difference of 10% between groups as the main analysis of TSA, since the adverse event rates reported were normally under 20% (Tan et al., 2019; Zhou et al., 2019). The estimated RIS allowed for a type I error of 0.05 and a type II error of 0.2 in the TSA analysis, and the analysis included the variance and heterogeneity of the effect estimates calculated by the random-effect model. We adjusted the significance boundaries by using the O’Brien-Fleming alpha-spending function, to ensure that the overall risk of type I error was within 5%.

## Results

### Characteristics of the Included Trials

**FIGURE 1:**
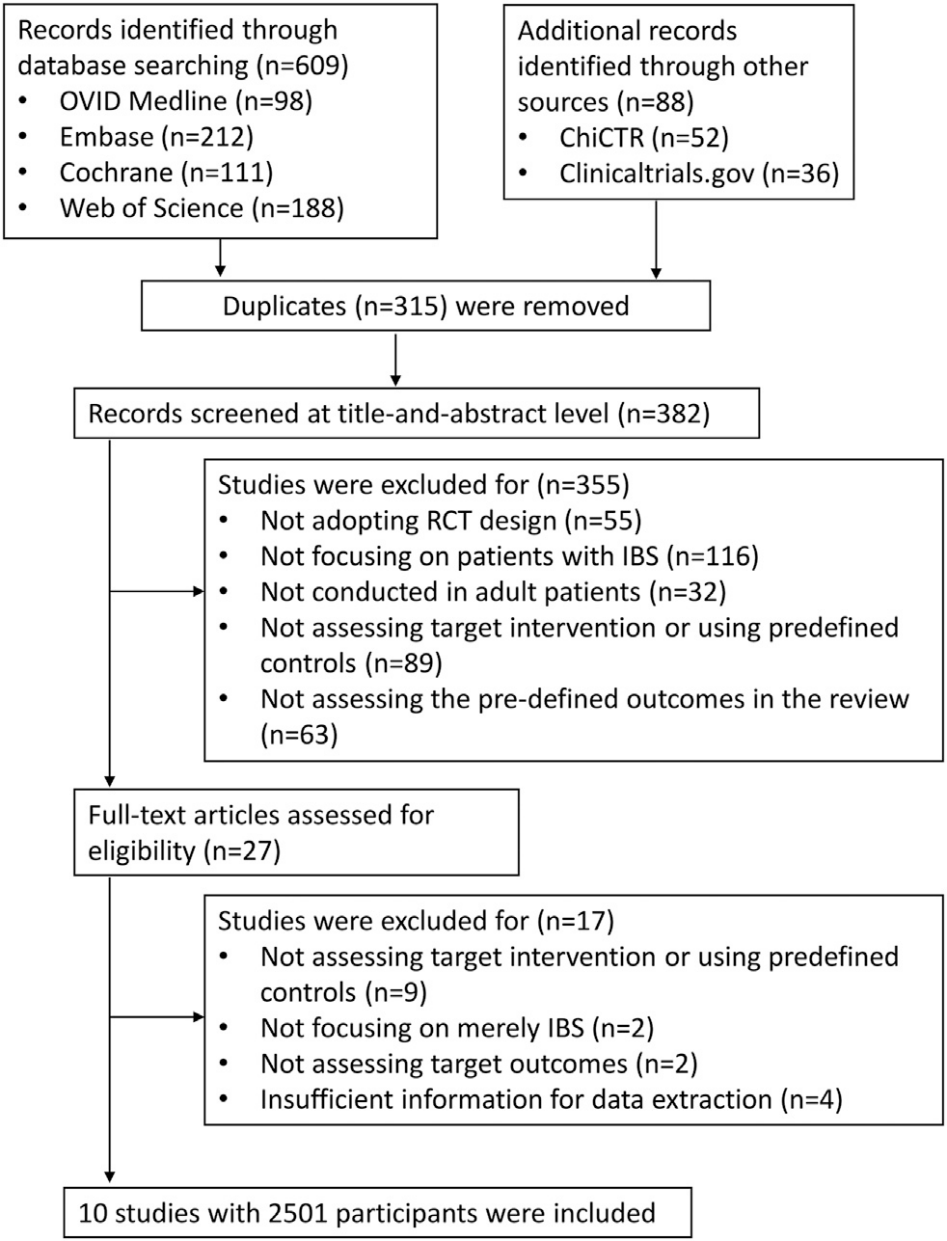
Study flowchart. The study flowchart shows the screening process of the study and results of each step.

Figure 1 shows the screening process of the systematic review. The search yields 697 records, and we finally included ten trials with 2,501 participants after screening.

Table 1 shows the characteristics of the included trials. The trials reported a median age of 43.9 years (ranged from 34 to 64 years). The proportion of female participants ranged from 41 to 93%. Six trials included participants with diarrhea-predominant IBS (Leung et al., 2006; Wang et al., 2006; Su et al., 2013; Chen et al., 2018; Tang et al., 2018; Tang et al., 2019), and the other five trials included all the subtypes of IBS (diarrhea-predominant IBS, constipation-predominant IBS, and mixed-type IBS) (Bensoussan et al., 1998, 2015; Fan et al., 2017; Shih et al., 2019).

**TABLE 1:** Characteristics of the included trials.

Author (publication year)	Design	Sample size	Mean age (year)	Female (%)	Diagnostic criteria	IBS subtype	Intervention	Treatment duration (week)	Efficacy outcomes
Bensoussan et al. (1998)	Multileft	116	48	0.45	Rome I	IBS-C, IBS-D, and IBS-M	Standardized or individualized CHM, 5 capsules tid	16	Relief of global IBS symptoms; IBS symptom scale
Bensoussan et al. (2015)	Multileft	125	48	0.93	Rome III	IBS-C, IBS-D, and IBS-M	Standardized CHM, 1 package tid	8	Relief of global IBS symptoms; IBS symptom scale; IBS-QOL
Chen et al. (2018)	Multileft	160	34	0.55	Rome III	IBS-D	Standardized CHM, 6 capsules tid	4	Relief of global IBS symptoms; relief of abdominal pain; IBS-related symptoms
Fan et al. (2017)	Multileft	1,044	36.5	0.5747	Rome III	IBS-C, IBS-D, and IBS-M	Standardized CHM, 400 ml tid	4	Abdominal pain; adequate relief of IBS symptoms
Leung et al. (2006)	Sing left	119	45	0.52	Rome II	IBS-D	Standardized CHM, 1 package bid	8	Relief of global IBS symptoms; abdominal pain; IBS-related symptoms; SF-36
Shih et al. (2019)	Single left	82	43	0.524	Rome III	IBS-C, IBS-D, and IBS-M	Standardized CHM, 3 g tid	4	Abdominal pain; SF-36
Su et al. (2013)	Multileft	240	37.5	57.5	Rome III	IBS-D	Standard CHM, bid	4	Relief of global IBS symptoms
Tang et al. (2018)	Multileft	216	43	0.41	Rome III	IBS-D	Standardized CHM, 1 package tid	8	Responder rate; adequate relief of global IBS symptoms; IBS-QOL
Tang et al. (2019)	Multileft	342	44.8	0.4795	Rome III	IBS-D	Standardized CHM, 5 g tid	6	Adequate relief of global IBS symptoms; HAMA; HAMD; IBS-QOL; IBS-SSS
Wang et al. (2006); Wang et al. (2018)	Single left	57	64	0.54	Rome II	IBS-D	Standardized CHM, 5 g tid	3	Relief of global IBS symptoms; abdominal pain

HAMA, Hamilton Anxiety Rating Scale. HAMD, Hamilton Depression Rating Scale. IBS-C, Constipation-predominant Irritable Bowel Syndrome. IBS-D, Diarrhea-predominant Irritable Bowel Syndrome. IBS-M, Mixed-type Irritable Bowel Syndrome. IBS-QOL, Irritable Bowel Syndrome Quality of Life instrument. IBS-SSS, Irritable Bowel Severity Scoring System. SF-36, 36-Item Short Form Survey.

Nine trials assessed the effect of standardized CHM (Sallon et al., 2002; Leung et al., 2006; Wang et al., 2006, 2018; Su et al., 2013; Bensoussan et al., 2015; Fan et al., 2017; Chen et al., 2018; Tang et al., 2018, 2019; Shih et al., 2019). The trials that included participants with diarrhea-predominant IBS used CHM prescriptions based on Tong-Xie-Yao-Fang or its revised version. Eight trials had a control group of placebo (Bensoussan et al., 1998; Bensooussan et al., 2015; Leung et al., 2006; Wang et al., 2006; Su et al., 2013; Chen et al., 2018; Shih et al., 2019; Tang et al., 2019), one trial had a control group of pinaverium (Tang et al., 2018), and one trial had control groups of both placebo and pinaverium (Fan et al., 2017). In summary, the interventions reported by the included trials were homogeneous in composition and treatment frequency. Table 2 shows the composition and dosage of CHM reported by the included trials.

**TABLE 2:** The composition and dosage of CHM reported in each trial.

First author (publication year)	The composition and dosage of CHM	Frequency of administration
Bensoussan et al. (1998)	*Codonopsis pilosula* (Franch.) Nannf. [Campanulaceae; Codonopsis Radix], 0.35 capsules	Standardized or individualized CHM, 5 capsules tid
	*Pogostemon cablin* (Blanco) Benth. [Lamiaceae; pogostemonis herba], 0.225 capsules	
	*Saposhnikovia divaricate* (Turcz. ex Ledeb.) Schischk. *Fraxinus chinensis* [Apiaceae; Saposhnikoviae Radix], 0.15 capsules	
	*Coix lacryma*-*jobi* L. var. ma-yuen (Roman.) Stapf. [Poaceae; Coicis Semen], 0.35 capsules	
	*Bupleurum chinense* DC. [Apiaceae; Bupleuri Radix], 0.225 capsules	
	*Artemisia capillaris* Thunb. [Asteraceae; Artemisiae Scopariae Herba], 0.65 capsules	
	*Atractylodes macrocephala* koidz. [Asteraceae; Atractylodis Macrocephalae Rhizoma], 0.45 capsules	
	*Magnolia officinalis* Rehd. et Wils. [Magnoliaceae; Magnoliae Officinalis Cortex], 0.225 capsules	
	*Citrus reticulata* Blanco [Rutaceae; Citri Reticulatae Pericarpium], 0.15 capsules	
	*Zingiber officinale* Rosc. [Zingiberaceae; Zingiberis Rhizoma Praeparatum], 0.225 capsules	
	*Fraxinus rhynchophylla* Hance [Oleaceae; Fraxini Cortex], 0.225 capsules	
	*Poria cocos* (Schw.) Wolf [Polyporaceae; Poria], 0.225 capsules	
	*Angelica dahurica* (Hoffm.) Benth. and Hook.f. ex Franch. and Sav. [Apiaceae; Angelicae Dahuricae Radix], 0.1 capsules	
	*Plantago asiatica* L. [Plantaginaceae; Plantaginis Semen], 0.225 capsules	
	*Phellodendron chinense* C.K.Schneid. [Rutaceae; Phellodendri Chinensis Cortex], 0.225 capsules	
	*Glycyrrhiza uralensis* Fisch. ex DC. [Fabaceae; Glycyrrhizae Radix et Rhizoma Praeparata Cum Melle], 0.225 capsules	
	*Paeonia lactiflora* Pall. [Paeoniaceae; Paeoniae Radix Alba], 0.15 capsules	
	*Aucklandia lappa* Decne. [Asteraceae; Aucklandiae Radix], 0.15 capsules	
	*Coptis chinensis* Franch. [Ranunculaceae; Coptidis Rhizoma], 0.15 capsules	
	*Schisandra chinensis* (Turcz.) Baill. [Schisandraceae; Schisandrae Chinensis Fructus], 0.35 capsules	
(Bensoussan et al. (2015)	*Paeonia lactiflora* Pall. [Paeoniaceae; Paeoniae Radix Alba], 0.483 g	Standardized CHM, 5 capsules bid
	*Citrus aurantium* L. [Rutaceae; Aurantii Fructus Immaturus], 0.42 g	
	*Magnolia officinalis* Rehd.et Wils. [Magnoliaceae; Magnoliae Officinalis Cortex], 0.3045 g	
	*Citrus reticulata* Blanco [Rutaceae; Citri Reticulatae Pericarpium], 0.3045 g	
	*Glycyrrhiza uralensis* Fisch. ex DC. [Fabaceae; Glycyrrhizae Radix et Rhizoma Praeparata Cum Melle], 0.231 g	
	*Rheum palmatum* L. [Polygonaceae; Rhei Radix et Rhizoma], 0.21 g	
	*Atractylodes lancea* (Thunb.) DC. [Asteraceae; Atractylodis Rhizoma], 0.147 g	
Chen et al. (2018)	*Paeonia lactiflora* Pall. [Paeoniaceae; Paeoniae Radix Alba], 6.7 g	Standardized CHM, 6 capsules tid
	*Saposhnikovia divaricate* (turcz.) Schischk. [Apiaceae; Saposhnikoviae Radix], 3.7 g	
	*Citrus reticulata* Blanco [Rutaceae; Citri Reticulatae Pericarpium], 5 g	
	*Atractylodes macrocephala* Koidz. [Asteraceae; Atractylodis Macrocephalae Rhizoma], 10 g	
Fan et al. (2017)	*Atractylodes macrocephala* Koidz. [Asteraceae; Atractylodis Macrocephalae Rhizoma]	Standardized CHM, 400 ml tid
	*Paeonia lactiflora* Pall. [Paeoniaceae; Paeoniae Radix Alba]	
	*Citrus reticulata* Blanco [Rutaceae; Citri Reticulatae Pericarpium]	
	*Saposhnikovia divaricate* (Turcz.) Schischk. [Apiaceae; Saposhnikoviae Radix]	
	*Codonopsis pilosula* (Franch.) Nannf. [Campanulaceae; Codonopsis Radix]	
	*Curcuma longa* L. [Zingiberaceae; Curcumae Radix]	
	*Citrus medica* L. [Rutaceae; Citri Sarcodactylis Fructus]	
	*Poria cocos* (Schw.) Wolf [Polyporaceae; Poria]	
	(Doses were not reported)	
Leung et al. (2006)	*Atractylodes macrocephala* Koidz. [Asteraceae; Atractylodis Macrocephalae Rhizoma], 15 g	Standardized CHM, 1 package bid
	*Astragalus mongholicus* Bunge [Fabaceae; Astragali Radix], 15 g	
	*Paeonia lactiflora* Pall. [Paeoniaceae; Paeoniae Radix Alba], 15 g	
	*Atractylodes lancea* (Thunb.) DC. [Asteraceae; Atractylodis Rhizoma], 12 g	
	*Bupleurum chinense* DC. [Apiaceae; Bupleuri Radix], 9 g	
	*Citrus reticulata* Blanco [Rutaceae; Citri Reticulatae Pericarpium], 9 g	
	*Saposhnikovia divaricate* (Turcz.) Schischk. [Apiaceae; Saposhnikoviae Radix], 9 g	
	*Murraya paniculata* (L.) Jack [Rutaceae; Murrayae Folium et Cacumen], 9 g	
	*Punica granatum* L. [Lythraceae; Granati Pericarpium] 9 g	
	*Portulaca oleracea* L. [Portulacaceae; Portulacae Herba], 30 g	
	*Coptis chinensis* Franch. [Ranunculaceae; Coptidis Rhizoma], 6 g	
Shih et al. (2019)	*Panax ginseng* C. A. Mey. [Araliaceae; Ginseng Radix et Rhizoma], 2.5 g	Standardized CHM, 3 g tid
	*Atractylodes macrocephala* Koidz. [Asteraceae; Atractylodis Macrocephalae Rhizoma], 5 g	
	*Poria cocos* (Schw.) Wolf [Polyporaceae; Poria], 5 g	
	*Glycyrrhiza uralensis* Fisch. ex DC. [Fabaceae; Glycyrrhizae Radix et Rhizoma Praeparata Cum Melle], 2 g	
	*Citrus reticulata* Blanco [Rutaceae; Citri Reticulatae Pericarpium], 2 g	
	*Pinellia ternata* (Thunb.) Breit. [Araceae; Pinelliae Rhizoma], 2.5 g	
	*Amomum villosum* Lour. [Zingiberaceae; Amomi Fructus], 2 g	
	*Aucklandia lappa* Decne. [Asteraceae; Aucklandiae Radix], 2 g	
	*Zingiber officinale* Rosc. [Zingiberaceae; Zingiberis Rhizoma Recens], 5 g	
(Su et al. (2013)	*Myristica fragrans* Houtt. [Myristicaceae; Myristicae Semen], 15 g	Standard CHM, bid
	*Psoralea corylifolia* L.[Fabaceae; Psoraleae Fructus], 30 g	
	*Schisandra chinensis* (Turcz.) Baill. [Schisandraceae; Schisandrae Chinensis Fructus], 9 g	
	*Tetradium ruticarpum* (A.Juss.) T.G.Hartley [Rutaceae; Euodiae Fructus], 9 g	
	*Codonopsis pilosula* (Franch.) Nannf. [Campanulaceae; Codonopsis Radix], 30 g	
	*Atractylodes macrocephala* Koidz. [Asteraceae; Atractylodis Macrocephalae Rhizoma], 15 g	
	*Curcuma wenyujin* Y. H. Chen et C. Ling [zingiberaceae; Curcumae Radix], 18 g	
	*Zingiber officinale* Rosc. [Zingiberaceae; Zingiberis Rhizoma Recens], 10 g	
Tang et al. (2018)	*Astragalus mongholicus* Bunge [Fabaceae; Astragali Radix], 18 g	Standardized CHM, 1 package tid
	*Atractylodes macrocephala* Koidz. [Asteraceae; Atractylodis Macrocephalae Rhizoma], 18 g	
	*Paeonia lactiflora* Pall. [Paeoniaceae; Paeoniae Radix Alba], 24 g	
	*Saposhnikovia divaricate* (Turcz.) Schischk. [Apiaceae; Saposhnikoviae Radix], 9 g	
	*Zingiber officinale* Rosc. [Zingiberaceae; Zingiberis Rhizoma Praeparatum], 6 g	
	*Myristica fragrans* Houtt. [Myristicaceae; Myristicae Semen], 9 g	
	*Pinellia ternata* (Thunb.) Breit. [Araceae; Pinelliae Rhizoma], 9 g	
	*Aucklandia lappa* Decne. [Asteraceae; Aucklandiae Radix], 12 g	
	*Citrus reticulata* Blanco [Rutaceae; Citri Reticulatae Pericarpium], 9 g	
	*Coptis chinensis* Franch. [Ranunculaceae; Coptidis Rhizoma], 6 g	
	*Glycyrrhiza uralensis* Fisch. ex DC. [Fabaceae; Glycyrrhizae Radix et Rhizoma Praeparata Cum Melle], 6 g	
Tang et al. (2019)	*Paeonia lactiflora* Pall. [Paeoniaceae; Paeoniae Radix Alba], 2.027 g	Standardized CHM, 5 g tid
	*Citrus reticulata* Blanco [Rutaceae; Citri Reticulatae Pericarpium Viride], 1.081 g	
	*Allium chinense* G. Don [Amaryllidaceae; Allii Macrostemonis Bulbus], 0.811 g	
	*Atractylodes macrocephala* Koidz. [Asteraceae; Atractylodis Macrocephalae Rhizoma], 1.081 g	
Wang et al. (2006); Wang et al. (2018)	*Paeonia lactiflora* Pall. [Paeoniaceae; Paeoniae Radix Alba], 2.027 g	Standardized CHM, 5 g tid
	*Citrus reticulata* Blanco [Rutaceae; Citri Reticulatae Pericarpium Viride], 1.081 g	
	*Allium chinense* G. Don [Amaryllidaceae; Allii Macrostemonis Bulbus], 0.811 g	
	*Atractylodes macrocephala* Koidz. [Asteraceae; Atractylodis Macrocephalae Rhizoma], 1.081 g	

The RoB assessment showed that seven trials were at low RoB (Bensoussan et al., 1998; Bensoussan et al., 2015; Leung et al., 2006; Fan et al., 2017; Chen et al., 2018; Tang et al., 2018; Shih et al., 2019) and the other three trials (Wang et al., 2006; Su et al., 2013; Tang et al., 2019) were with some concerns. Two trials have rated some concerns in the domain of deviations from intended interventions, one trial has rated some concerns in the domain of measurement of the outcome, and two trials were rated with some concerns in the domain of selection of the reported results. The details of the RoB assessment are shown in the supplementary files (Supplementary Figure 1).

### Adequate Relief of Global Symptoms of IBS

#### Comparison With Placebo

**FIGURE 2:**
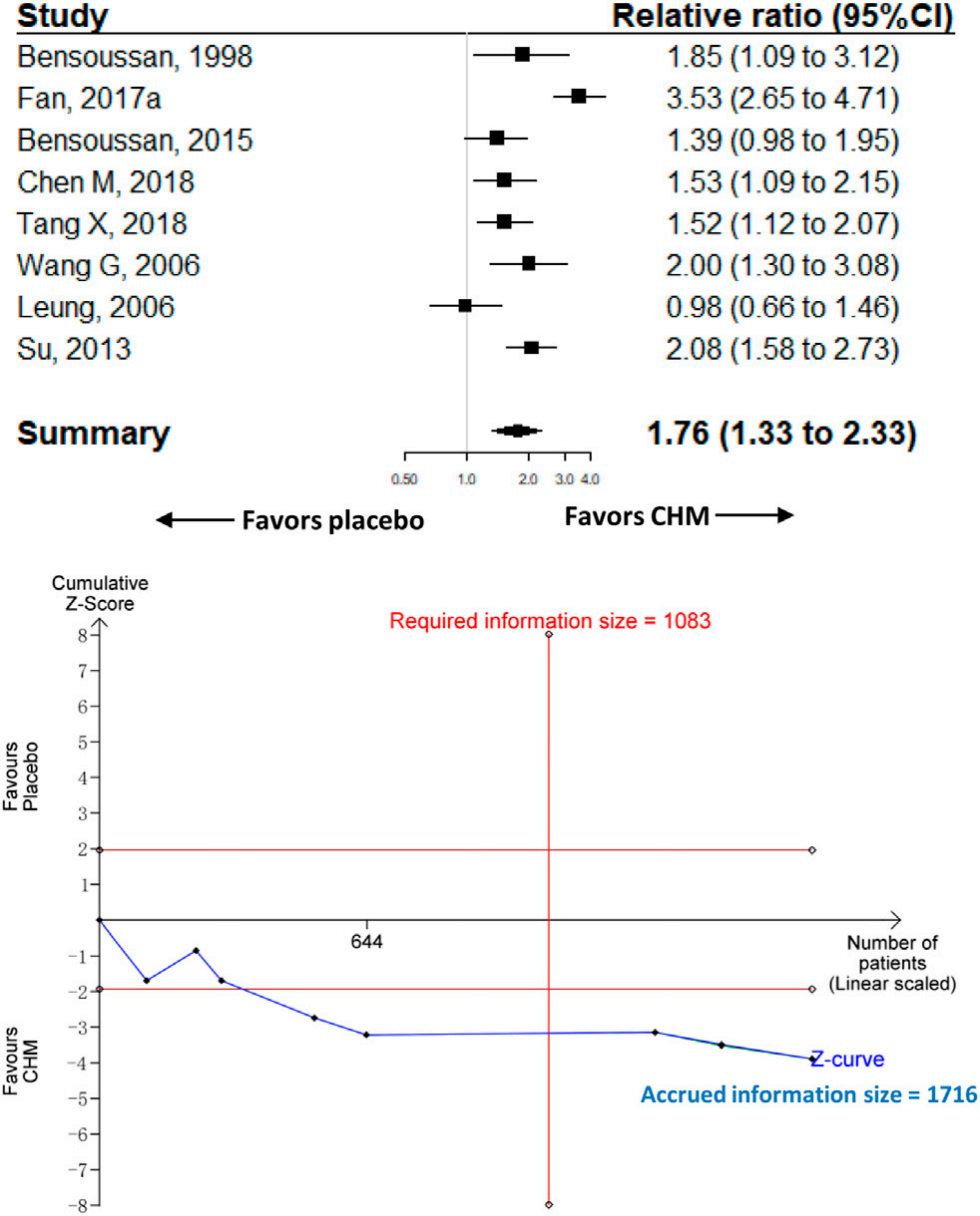
Meta-analysis and TSA analysis of CHM vs. placebo in the adequate relief of global IBS symptoms. CHM, Chinese herbal medicine. TSA, trial sequential analysis. The results of meta-analysis were shown on the top half, and the results of TSA were showed on the bottom half. The red vertical line represented the required information size (RIS) for the comparison, assuming a difference of 20% in the proportion of adequate relief of global IBS symptoms. The blue line represented the cumulative Z curve, and the black dots represented the included studies added according to their publication year in ascending order.

Eight trials with 1,716 participants were included to compare the effect of CHM (*n* = 885) with placebo (*n* = 831) on adequate relief of global IBS symptoms. Participants in the CHM group had significantly a higher proportion of adequate relief [RR 1.76 (95%CI, 1.33–2.33); *I* ^*2*^ = 81.1%; *p* < 0.001] (Figure 2). Contour-enhanced funnel plot revealed no significant publication bias (Supplementary Figure 2). The trim-and-fill analysis showed consistent results. The sensitivity analysis found that one trial (Fan et al., 2017) had a significant impact on the heterogeneity of the meta-analysis; after removing the trial, the *I* ^*2*^ dropped to 47.9%, and we reran the meta-analysis and found similar results but with smaller effect size than the main analysis (RR, 1.58 [95%CI, 1.31 to 1.91]; *p* < 0.001) (Supplementary Figure 3).

#### Comparison With Pinaverium

Two trials with 1,032 participants were included to compare CHM (*n* = 516) with pinaverium (*n* = 516), and the results showed that participants taking CHM had a slightly higher rate of adequate relief of global IBS symptoms compared with pinaverium [RR, 1.10 (95%CI, 0.99 to 1.22); *I* ^*2*^ = 49%; *p* = 0.16] (Supplementary Figure 4).

#### TSA

The TSA analysis showed that, assuming a 20% difference between CHM and placebo in the proportion of adequate relief of global IBS symptoms, the RIS required 1,083 participants and the accrued sample size (*n* = 1,716) of this meta-analysis exceeded the RIS. Figure 2 shows that the cumulative Z curve crossed trial sequential boundaries, indicating a statistically significant difference between CHM and placebo. Supplementary Figures 5, 6 show the results of another two TSA analyses that assumed the between-group difference of 15 and 10% in the proportion of adequate relief of global IBS symptoms, respectively.

### Relief of Abdominal Pain

#### Comparison With Placebo

**FIGURE 3:**
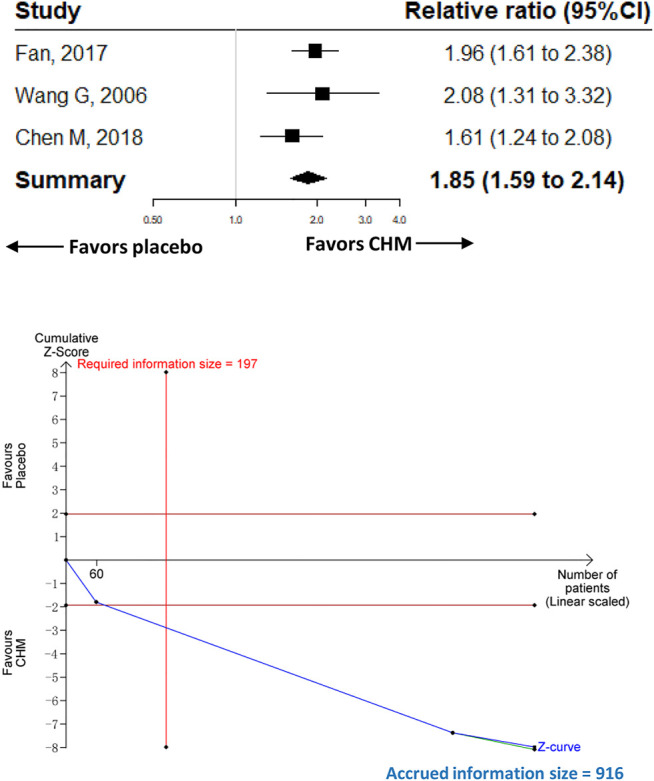
Meta-analysis and TSA analysis of CHM vs. placebo in the relief of abdominal pain. CHM, Chinese herbal medicine. TSA, trial sequential analysis. The results of meta-analysis were shown on the top half, and the results of TSA were shown on the bottom half. The red vertical line represented the RIS for the comparison, assuming a difference of 20% in the rate of the relief of abdominal pain. The blue line represented the cumulative Z curve, and the black dots represented the included studies added according to their publication year in ascending order.

Three trials with 916 participants were included to compare the effect of CHM (*n* = 458) with placebo (*n* = 458) on abdominal pain. The meta-analysis of the three trials showed that participants receiving CHM had a higher proportion of abdominal pain relief than those receiving placebo [RR 1.85 (95%CI, 1.59–2.14); *I* ^*2*^ = 0%; *p* < 0.001] (Figure 3). Contour-enhanced funnel plot revealed no publication bias. The trim-and-fill analysis and sensitivity analysis showed the same results as the main analysis.

#### TSA

The TSA analysis was performed based on the comparison of CHM vs. placebo, and the analysis showed that the RIS required 197 participants when we assumed a 20% difference in the proportion of relief of abdominal pain, and the cumulative Z curve crossed trial sequential boundaries, indicating a statistically significant difference between CHM and placebo (Figure 3).

### Adverse Events

The most common adverse events were gastrointestinal disorders (e.g., nausea, constipation, diarrhea, or bloating), which were reported by 7 RCTs (Bensoussan et al., 1998, 2015; Wang et al., 2006; Fan et al., 2017; Chen et al., 2018; Tang et al., 2018, 2019); these gastrointestinal disorders were mild and cured without additional treatment. Two trials reported skin rash (Chen et al., 2018; Tang et al., 2019) and three reported elevated liver enzyme (Chen et al., 2018; Tang et al., 2018, 2019); these adverse events were all mild.

#### Comparison With Placebo

**FIGURE 4:**
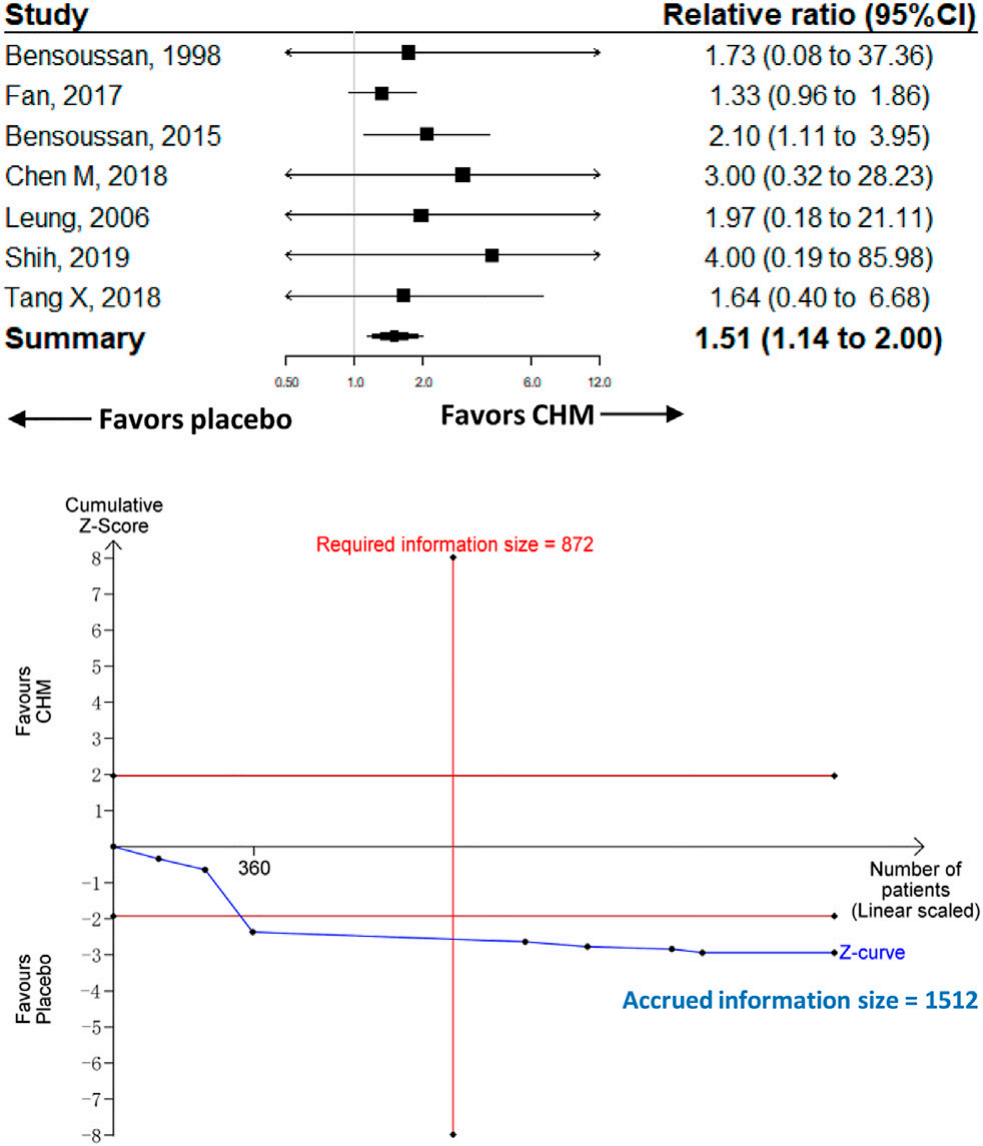
Meta-analysis and TSA analysis of CHM vs. placebo in the treatment-related adverse events. CHM, Chinese herbal medicine. TSA, trial sequential analysis. The results of meta-analysis were shown on the top half, and the results of TSA were shown on the bottom half. The red vertical line represented the RIS for the comparison, assuming a difference of 5% in the rate of treatment-related adverse events. The blue line represented the cumulative Z curve, and the black dots represented the included studies added according to their publication year in ascending order.

Seven trials recruiting 1,512 participants were included in the comparison of CHM (*n* = 779) vs. placebo (*n* = 733). The meta-analysis showed that CHM was associated with a significantly higher proportion of adverse events compared with placebo [RR 1.51 (95%CI, 1.14–2); *I* ^*2*^ = 0%; *p* = 0.004] (Figure 4). The contour-enhanced funnel plot showed no evidence of publication bias, and the sensitivity analysis showed consistent results with the main analysis (Supplementary Figure 7).

#### Comparison With Pinaverium

Two trials recruiting 1,032 participants were included in the comparison of CHM (*n* = 516) vs. pinaverium (*n* = 516). The meta-analysis showed that the proportion of adverse events was similar between CHM and pinaverium [CHM vs. pinaverium, RR 1.06 (95%CI, 0.78–1.42); *I* ^*2*^ = 0%; *p* = 0.79] (Supplementary Figure 8).

#### TSA

The TSA analysis was performed based on the comparison of CHM vs. placebo, and the analysis showed that the RIS was 872 participants when we considered a 10% difference in the proportion of adverse events (Figure 4).

### Subgroup Analysis

The subgroup analysis including participants with diarrhea-predominant IBS only showed results consistent with the main analysis. CHM was associated with a higher rate of adequate relief of global IBS symptoms compared with placebo [6 trials with 1,475 participants; RR 1.81 (95%CI, 1.28–2.58); *I* ^*2*^ = 85%; *p* < 0.001], and it was associated with a higher rate of adverse events compared with placebo [five trials with 1,527 participants; RR 1.37 (95%CI, 1.01–1.86); *I* ^*2*^ = 0%; *p* = 0.039]. Supplementary Figures 9, 10 show the forest plots of subgroup analysis.

## Discussion

### Main Findings

Whether CHM is effective and safe in the management of IBS is still under investigation, according to the 2019 Canadian Association of Gastroenterology Clinical Practice Guideline (Moayyedi et al., 2019). Our meta-analysis showed that CHM was superior over placebo in relieving global IBS symptoms and abdominal pain, and CHM had a similar effect as pinaverium in relieving abdominal pain, which indicated that CHM is effective as a treatment for IBS. These findings were further confirmed by the TSA analysis, which showed that our meta-analyses had a sufficient sample size to determine a 20% difference in adequate relief of IBS symptoms and abdominal pain.

### Strength of the Study

Our study has several strengths. First, we confirmed that the RIS was reached in the outcomes of adequate relief of global IBS symptoms and abdominal pain for measuring the effect of CHM on IBS, which provided evidence from another perspective that the positive findings regarding the effect of CHM were unlikely to be false-positive (type I error). Second, we used Cochrane RoB 2.0 to assess the RoB in the included trials. Compared with its previous versions, RoB 2.0 had several advantages: providing an evaluation of the overall RoB for an individual study, using fixed RoB domains (avoiding inconsistent use of RoB: removing or adding domains when performing assessment), providing signaling questions to facilitate judgment, and using “some concerns” instead of “unclear RoB ” to avoid overuse of “unclear risk” in RoB assessment. Third, we performed trim-and-fill analysis, sensitivity analysis, and subgroup analysis to ensure the robustness of the study findings.

### Comparison With Other Studies

Previous systematic reviews showed a beneficial effect of CHM on IBS. Our review provided more convincing evidence. First, we abandoned searching the studies published in Chinese databases (e.g., the China National Knowledge Infrastructure), since these studies normally adopted the outcome—clinically effective rate, which had varied definitions—which might cause undetectable heterogeneity in the meta-analysis. Additionally, most of these studies tend to be rated with a high RoB, including them in the systematic review would decrease the certainty of the evidence. Second, we used TSA analysis to control for false-positive findings. Third, we used more stringent and clearer criteria for determining the degree of heterogeneity. Previous meta-analyses adopted a *I* ^*2*^ < 50% as low heterogeneity (Tan et al., 2019; Zhou et al., 2019), while our study treated an *I* ^*2*^ between 30 and 60% as moderate heterogeneity, as stated in Cochrane Handbook 5.1. However, there is a common problem that was still unsolved in our review; the long-term effect of CHM (a follow-up period longer than 3 months) has not been assessed owing to the lack of original studies. Fourth, our study added several more recently published RCTs (especially those published in 2018 and 2019), which had larger sample sizes than previous RCTs.

### Implications for Clinical Practice

We found that, in most RCTs recruiting only patients with diarrhea-predominant IBS, Tong-Xie-Yao-Fang was commonly used and consisted of four herbal components: *Radix Paeoniae Alba* (Baishao), *Radix Saposhnikoviae* (Fangfeng), *Pericarpium Citri Reticulate* (Chenpi), and *Rhizoma Atractylodis Macrocephalae* (Baizhu). The mechanism of the effect of Tong-Xie-Yao-Fang on diarrhea-predominant IBS has been demonstrated in several studies, which includes adjusting mast cell activation to decrease visceral hypersensitivity (Pan et al., 2009), regulating the brain-gut axis through decreasing serotonin levels in serum and brain concentrations of corticotrophin-releasing factor (Hu et al., 2009), inhibiting colon contraction through inhibition of extracellular calcium internal flow (Yang et al., 2015), relieving visceral hypersensitivity through regulating brain-derived neurotrophic factor (Chen et al., 2017), and diminishing colonic serotonin levels though normalizing gut flora (Li et al., 2018). These findings suggested that future studies should focus on assessing the effect of Tong-Xie-Yao-Fang on diarrhea-predominant IBS.

The adverse effect of CHM was also a major concern as shown by our study, which demonstrated a significantly higher proportion of adverse events in CHM administration compared with placebo. However, this might not prevent CHM from clinical practice, since all of the adverse events were mild and CHM had only a slightly higher adverse event rate than pinaverium. The high tolerance of pinaverium in clinical practice indicates also a possible acceptance of CHM for IBS in routine practice. Additionally, with further clarification of the mechanism of the effect of CHM on IBS, the adverse effect will be recognized more clearly and might lead to a decrease in adverse event rate.

### Study Limitations

Our study is not exempt from limitations. First, we did not search Chinese databases, which included a large number of studies on the effectiveness of CHM. However, including these studies in our meta-analysis may increase the RoB. In addition, we limited the literature searching at the start of the year 1980, because previously published systematic reviews and meta-analyses of CHM showed that all the included studies were published after 1980, we assumed that the retrieving of literature before 1980 would increase the burden of screening but provide no additional benefit. As the development of evidence-based medicine after the 1990s, especially in the new millennium, the quality of clinical trials is prominently improved, and we contemplated that pooling the results of trials conducted in a wide time span (>40 years) would lead to significant heterogeneity in study populations, interventions, and outcome measures. Second, clinical heterogeneity in the preparation of CHM could not be detected in the statistical models of our study. Third, several included RCTs recruited participants with all subtypes of IBS; although it increased the generalizability of the study results, it made the results difficult to explain. However, we ran a subgroup analysis by including participants with diarrhea-predominant IBS only, and the result was consistent with the main analysis. Fourth, we did not register our protocol prior to the implementation of our study, which might cause overlapping work. The reasons for unregistered protocol were that the registration time during the COVID-19 pandemic is longer than usual and we wanted to inform investigators in time for whether the clinical question of the effectiveness of CHM for IBS was answered with sufficient statistical power.

## Conclusion

Our study showed that CHM was effective in relieving global IBS symptoms and abdominal pain but was associated with a higher rate of adverse events compared with placebo. TSA analysis confirmed the findings of our meta-analysis. Regarding that most of the adverse events were mild and cured without the need for additional medical care, we asserted that CHM might be a potential candidate for patients with IBS.

## Abbreviations

95%CIs, 95% confidence intervals; CAMs, complementary and alternative medicines; CHM, Chinese herbal medicine; FDA, Food and Drug Administration; IBS, irritable bowel syndrome; RCTs, randomized controlled trials; RIS, required information size; RoB, risk of bias; RR, relative ratio; TSA, trial sequential analysis; VAS, visual analog scale.
