# Supplementary material for: Chinese Herbal Medicine for Irritable Bowel Syndrome: A Meta-Analysis and Trial Sequential Analysis of Randomized Controlled Trials
Source: Front Pharmacol. 2021 Jul 27;12:694741. doi: 10.3389/fphar.2021.694741 (PMC8353248; doi:10.3389/fphar.2021.694741)
Supplement: Supplementary file 1 [file DataSheet1.docx]

**Supplementary files**

[sTable 1. Search strategy in OVID Medline 2](#_Toc75462423)

[sTable 2. Search strategy in Embase 3](#_Toc75462424)

[sTable 3. Search strategy in CENTRAL 4](#_Toc75462425)

[sTable 4. Search strategy in Web of Science 5](#_Toc75462426)

[sFigure 1. Overall risk of bias 6](#_Toc75462427)

[sFigure 2. Contour-enhanced funnel plot for the analysis of global IBS symptoms 7](#_Toc75462428)

[sFigure 3. Leave-one-out analysis for the analysis of global IBS symptoms 8](#_Toc75462429)

[sFigure 4. Comparison with pinaverium for the analysis of global IBS symptoms 8](#_Toc75462430)

[sFigure 5. TSA analysis for global IBS symptoms assuming a 15% difference between groups 9](#_Toc75462431)

[sFigure 6. TSA analysis for global IBS symptoms assuming a 10% difference between groups 9](#_Toc75462432)

[sFigure 7. Sensitivity analysis for the analysis of adverse events 10](#_Toc75462433)

[sFigure 8. Comparison with pinaverium for the analysis of adverse events 10](#_Toc75462434)

[sFigure 9. Subgroup analysis of adequate relief of global IBS symptoms 11](#_Toc75462435)

[sFigure 10. Subgroup analysis of treatment-related adverse events 11](#_Toc75462436)

# sTable 1. Search strategy in OVID Medline

| **ID** | **Search strategy** |
| --- | --- |
| 1 | randomised controlled trial.pt. |
| 2 | randomized controlled trial.pt. |
| 3 | controlled clinical trial.pt. |
| 4 | randomized.ab. |
| 5 | randomised.ab. |
| 6 | randomly.ab. |
| 7 | or/1-6 |
| 8 | limit 7 to humans |
| 9 | exp Irritable Bowel Syndrome/ |
| 10 | irritable bowel syndrome. ti,ab. |
| 11 | IBS. ti,ab. |
| 12 | IBS$. ti,ab. |
| 13 | or/9-12 |
| 14 | exp herbal medicine/ |
| 15 | herbal medicine.ab. |
| 16 | exp phytotherapy/ |
| 17 | phytotherapy.ab. |
| 18 | exp plants, medicinal / |
| 19 | plants, medicinal.ab. |
| 20 | exp medicine, traditional/ |
| 21 | medicine,traditional.ab. |
| 22 | Chinese herb$.ab. |
| 23 | or/14-22 |
| 24 | 7 and 13 and 23 |

# sTable 2. Search strategy in Embase

| **ID** | **Search strategy** |
| --- | --- |
| 1 | ‘randomized Controlled Trial’/exp |
| 2 | ‘randomized Controlled Trials as Topic’/exp |
| 3 | ‘randomized controlled trial’:ab,ti |
| 4 | ‘controlled clinical trial’/exp |
| 5 | ‘controlled clinical trial’:ab,ti |
| 6 | (#1 OR #2 OR #3 OR #4 OR #5) AND [humans]/lim |
| 7 | ‘Irritable Bowel Syndrome’/exp |
| 8 | ‘irritable bowel syndrome’:ab,ti |
| 9 | ‘IBS’:ab,ti |
| 10 | ‘IBS$’:ab,ti |
| 11 | #7 OR #8 OR #9 OR #10 |
| 12 | ‘herbal medicine’/exp |
| 13 | ‘herbal medicine’:ab,ti |
| 14 | ‘phytotherapy’/exp |
| 15 | ‘phytotherapy’:ab,ti |
| 16 | ‘plants, medicinal’/exp |
| 17 | ‘plants, medicinal’:ab,ti |
| 18 | ‘medicine,traditional’/exp |
| 19 | ‘Chinese herb*’:ab,ti |
| 20 | #12 OR #13 OR #14 OR #15 OR #16 OR #17 OR #18 OR #19 |
| 21 | #6 AND #11 AND #20 |

# sTable 3. Search strategy in CENTRAL

| **ID** | **Search** |
| --- | --- |
| #1 | MeSH descriptor: [Randomized Controlled Trial] explode all trees |
| #2 | (randomized controlled trial):ti,ab,kw |
| #3 | (double blind):ti,ab,kw |
| #4 | (placebo):ti,ab,kw |
| #5 | #1 or #2 or #3 or #4 |
| #6 | MeSH descriptor: [Irritable Bowel Syndrome] explode all trees |
| #7 | MeSH descriptor: [Colonic Diseases, Functional] explode all trees |
| #8 | (irritable bowel syndrome*):ti,ab,kw |
| #9 | (IBS):ti,ab,kw |
| #10 | (functional abdominal pain):ti,ab,kw |
| #11 | (functional gastrointestinal disorder*):ti,ab,kw |
| #12 | #6 or #7 or #8 or #9 or #10 or #11 |
| #13 | MeSH descriptor: [Medicine, Chinese Traditional] explode all trees |
| #14 | MeSH descriptor: [Drugs, Chinese Herbal] explode all trees |
| #15 | MeSH descriptor: [Medicine, Kampo] explode all trees |
| #16 | (herb* medicine):ti,ab,kw |
| #17 | (tongxie*):ti,ab,kw |
| #18 | #13 or #14 or #15 or #16 or #17 |
| #19 | #5 and #12 and #18 |

# sTable 4. Search strategy in Web of Science

| **ID** | **Search strategy** |
| --- | --- |
| #1 | TS=(irritable bowel syndrome* or IBS or functional gastrointestinal disorder* or functional abdominal pain) or TI=(irritable bowel syndrome* or IBS or functional gastrointestinal disorder* or functional abdominal pain) or AB=(irritable bowel syndrome* or IBS or functional gastrointestinal disorder* or functional abdominal pain) |
| #2 | TS=(randomized controlled trial or placebo or double blind) or TI=(randomized controlled trial or placebo or double blind) or AB=(randomized controlled trial or placebo or double blind) |
| #3 | TS=(chinese medicin* or herb* or eluxadoline) or TI=(chinese medicin* or herb* or eluxadoline) or AB=(chinese medicin* or herb* or eluxadoline) |
| #4 | #3 AND #2 AND #1 |

# sFigure 1. Overall risk of bias


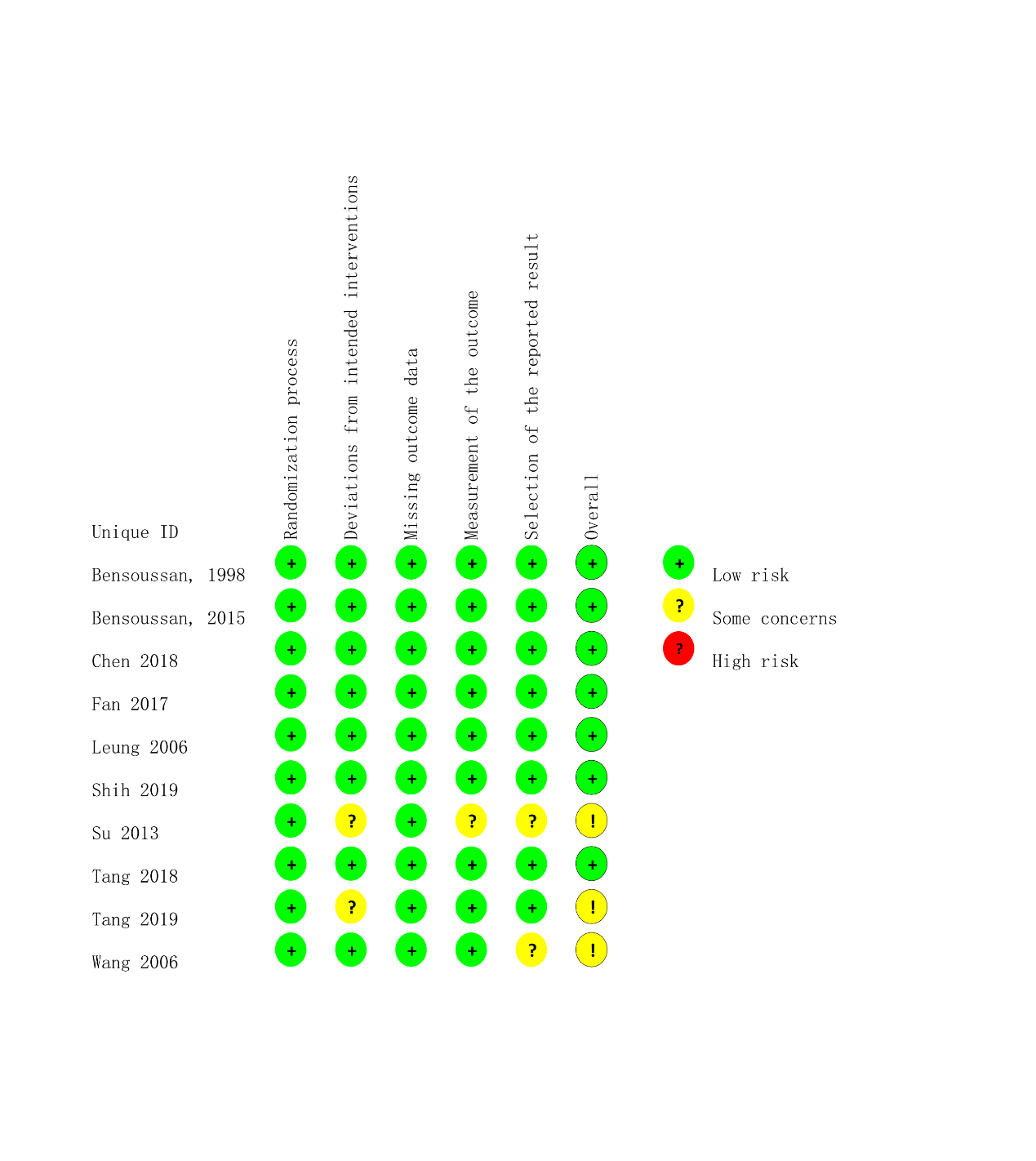


sFigure 1 shows the RoB assessment within each trial and the overall RoB.

# sFigure 2. Contour-enhanced funnel plot for the analysis of global IBS symptoms


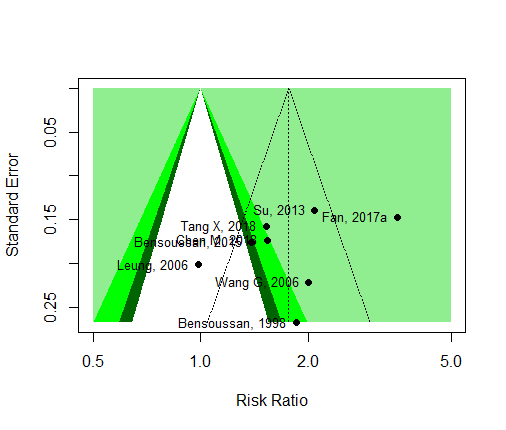


# sFigure 3. Leave-one-out analysis for the analysis of global IBS symptoms


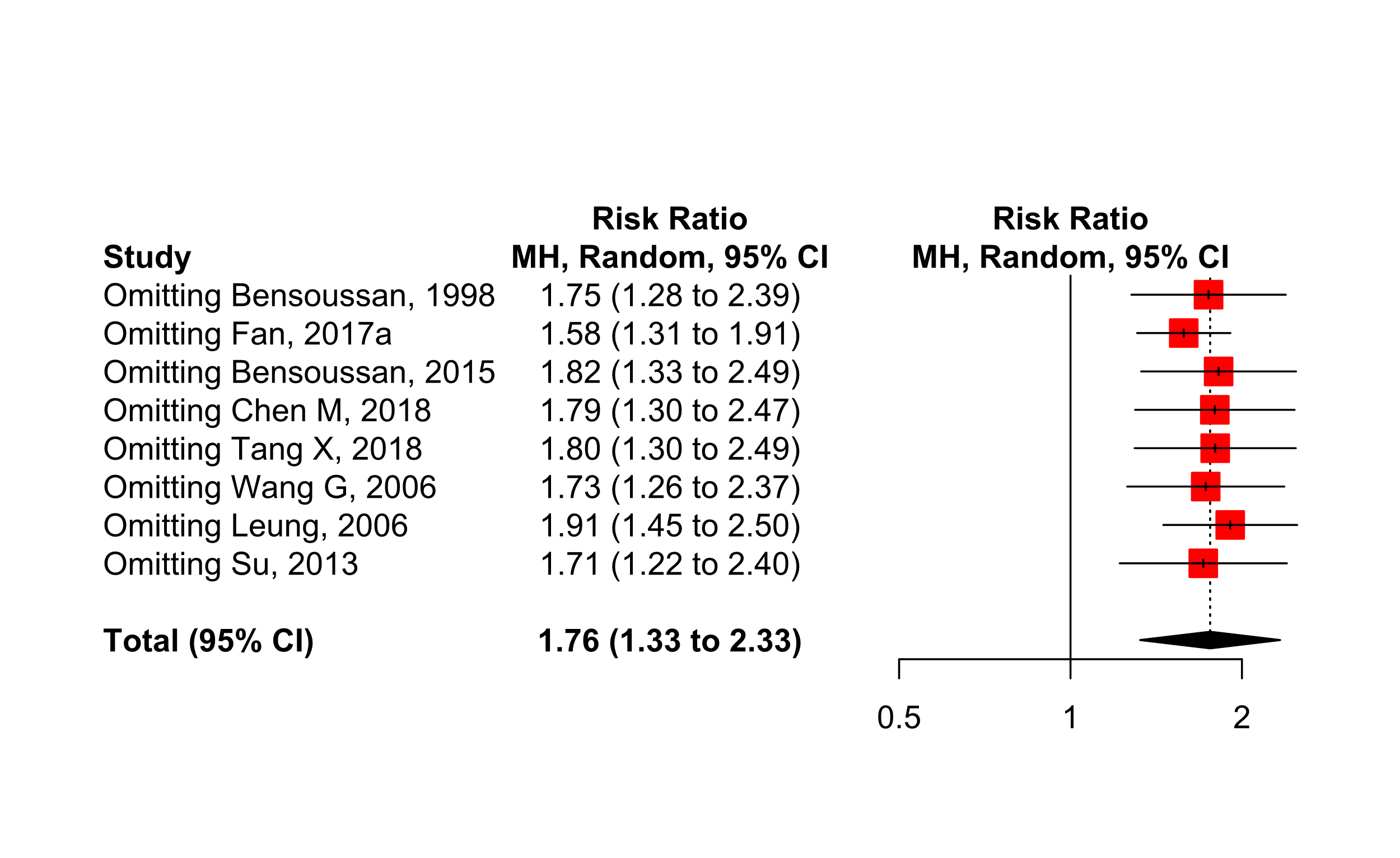


After omitting the study (Fan, 2017a), the *I^2^* statistics decreased to 47.9%.

# sFigure 4. Comparison with pinaverium for the analysis of global IBS symptoms


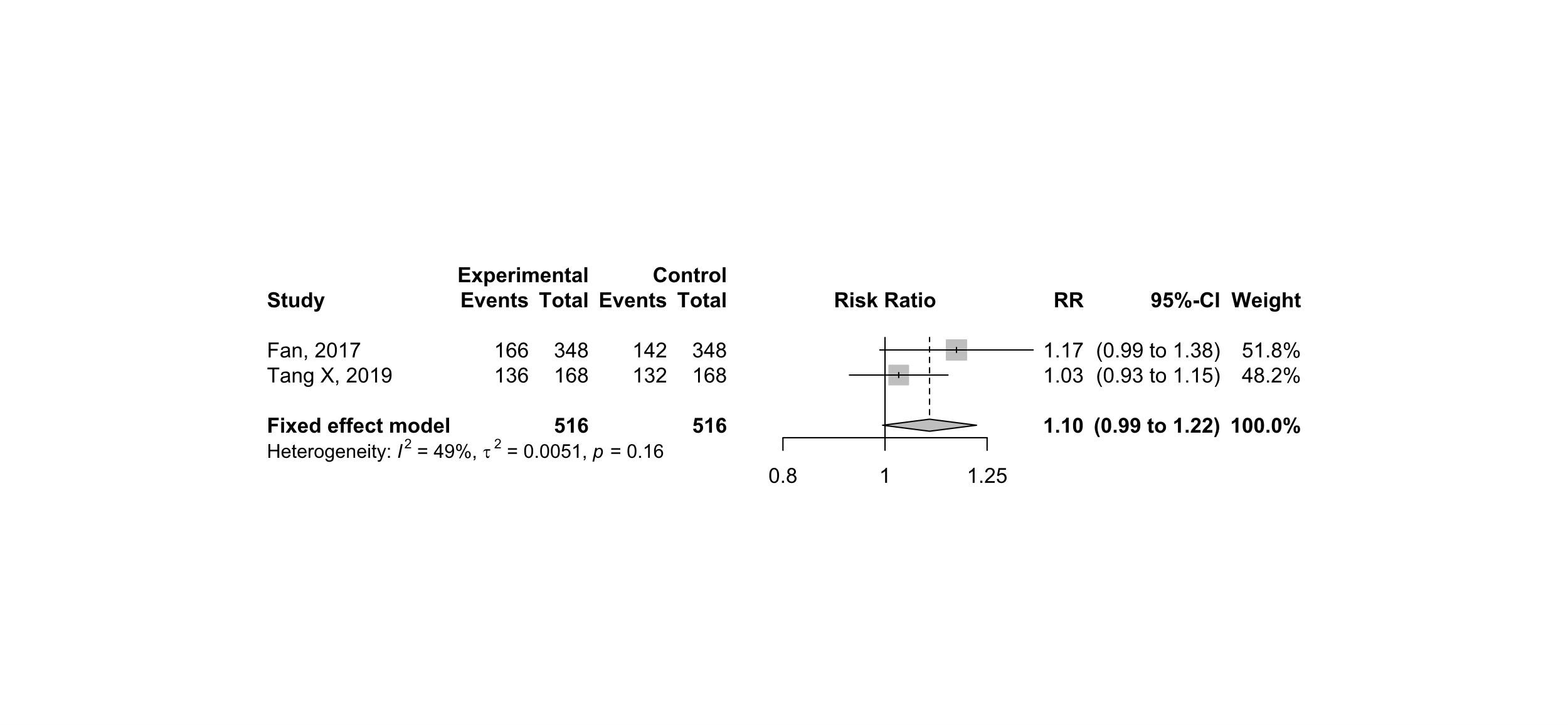


# sFigure 5. TSA analysis for global IBS symptoms assuming a 15% difference between groups


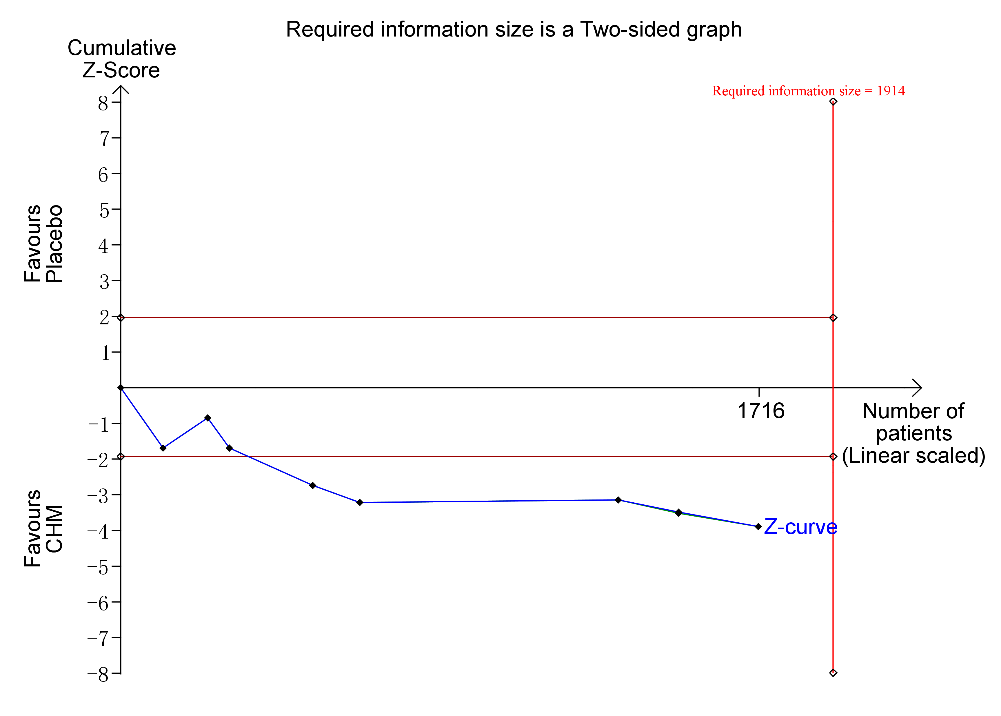


# sFigure 6. TSA analysis for global IBS symptoms assuming a 10% difference between groups


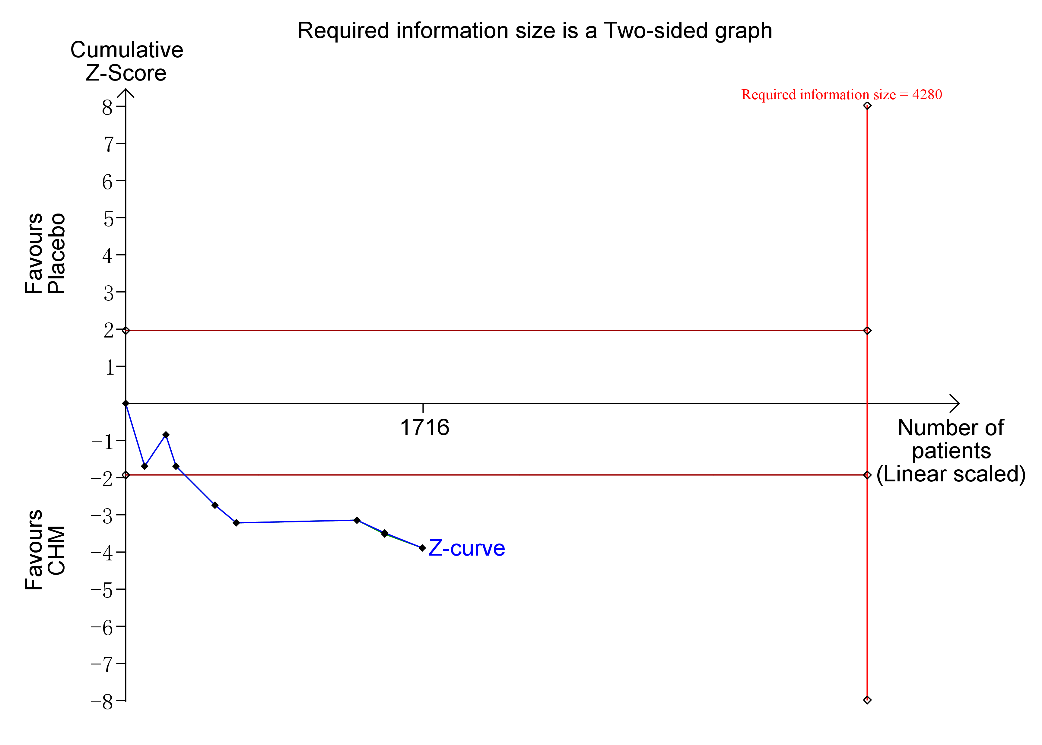


# sFigure 7. Sensitivity analysis for the analysis of adverse events


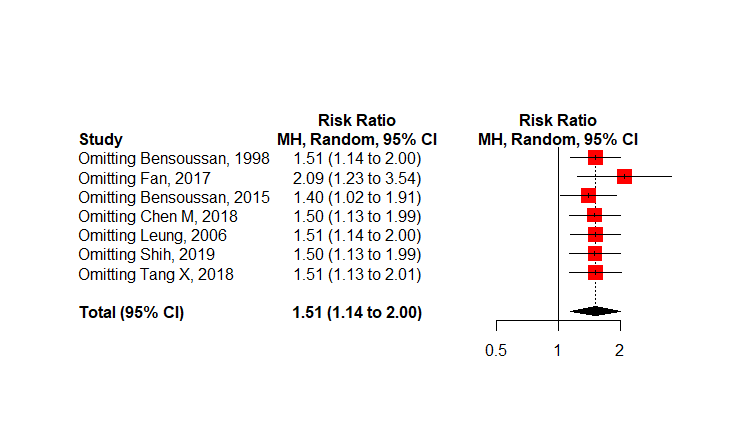


Leave-one-out analysis was performed as sensitivity analysis through omitting one study at at time and re-performing the analysis.

# sFigure 8. Comparison with pinaverium for the analysis of adverse events


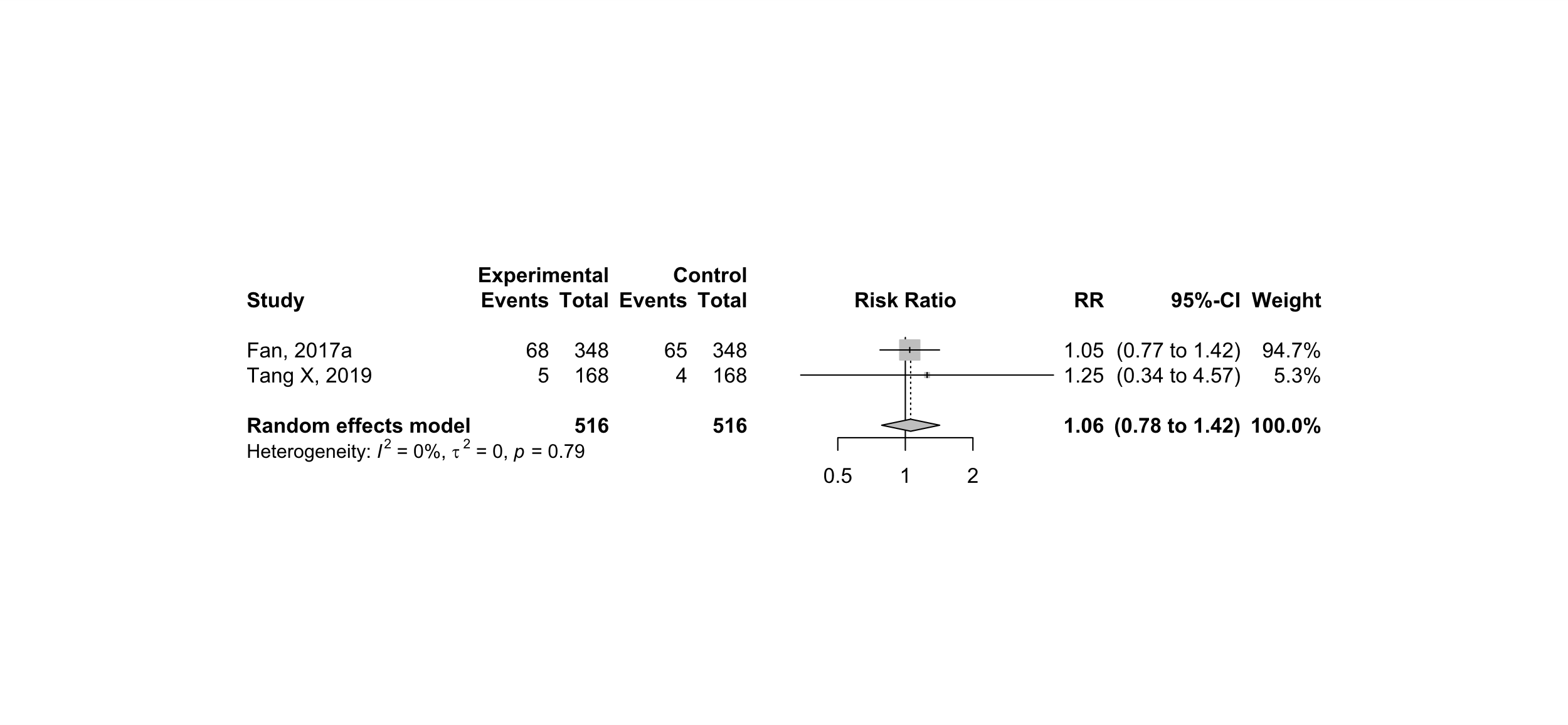


# sFigure 9. Subgroup analysis of adequate relief of global IBS symptoms


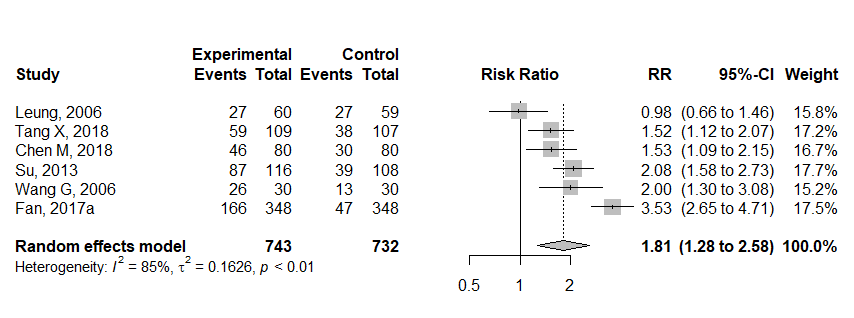


Experimental group was CHM, and the control group was placebo.

# sFigure 10. Subgroup analysis of treatment-related adverse events


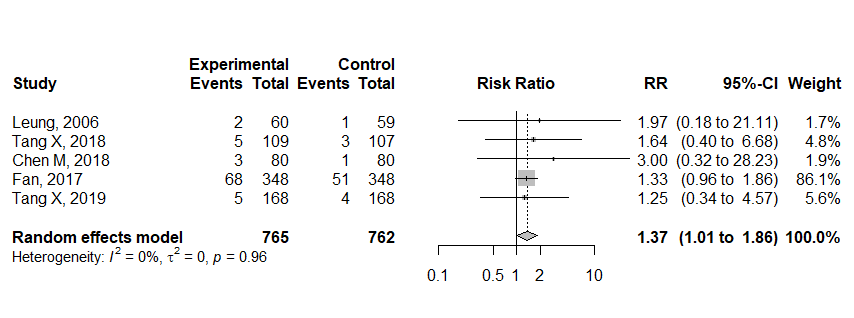


Experimental group was CHM, and the control group was placebo.
